# Optimisation of Prime–Boost Immunization in Mice Using Novel Protein-Based and Recombinant Vaccinia (Tiantan)-Based HBV Vaccine

**DOI:** 10.1371/journal.pone.0043730

**Published:** 2012-09-06

**Authors:** Hong Chen, Xia Chuai, Yao Deng, Bo Wen, Wen Wang, Shaoqing Xiong, Li Ruan, Wenjie Tan

**Affiliations:** 1 Biotech Center for Viral Diseases Emergency, National Institute for Viral Disease Control and Prevention, Chinese Center for Disease Control and Prevention, Beijing, People's Republic of China; 2 Department of Microbiology, Hebei Medical University, Shijiazhuang, People's Republic of China; 3 College of Life Science, Jilin University, Changchun, People's Republic of China; 4 School of Life Science and Bio-engineering, Beijing University of Technology, Beijing, People's Republic of China; Duke University Medical Center, United States of America

## Abstract

**Background:**

A therapeutic vaccine for chronic hepatitis B virus (HBV) infection that enhances virus-specific cellular immune responses is urgently needed. The “prime–boost” regimen is a widely used vaccine strategy against many persistence infections. However, few reports have addressed this strategy applying for HBV therapeutic vaccine development.

**Methodology/Principal Findings:**

To develop an effective HBV therapeutic vaccine, we constructed a recombinant vaccinia virus (Tiantan) containing the S+PreS1 fusion antigen (RVJSS1) combined with the HBV particle-like subunit vaccine HBVSS1 to explore the most effective prime–boost regimen against HBV. The immune responses to different prime–boost regimens were assessed in C57BL/C mice by ELISA, ELISpot assay and Intracellular cytokine staining analysis. Among the combinations tested, an HBV protein particle vaccine priming and recombinant vaccinia virus boosting strategy accelerated specific seroconversion and produced high antibody (anti-PreS1, anti-S antibody) titres as well as the strongest multi-antigen (PreS1, and S)-specific cellular immune response. HBSS1 protein prime/RVJSS1 boost immunization was also generated more significant level of both CD4+ and CD8+ T cell responses for Th1 cytokines (TNF-α and IFN-γ).

**Conclusions:**

The HBSS1 protein-vaccine prime plus RVJSS1 vector boost elicits specific antibody as well as CD4 and CD8 cells secreting Th1-like cytokines, and these immune responses may be important parameters for the future HBV therapeutic vaccines.

## Introduction

Hepatitis B virus (HBV) infection is a major global health problem. An estimated 2 billion people worldwide have been infected with the virus, and approximately 350 million are chronically infected that may lead to liver cirrhosis and hepatocellular carcinoma, causing 600,000 deaths per year [1]. Since the early 1980s, various types of HBV vaccine have been developed and contributed significantly to a decrease in the number of chronic HBV carriers [2]–[4]. The currently available recombinant protein vaccines for HBV, either expressed by yeast or Chinese hamster ovary (CHO) cells [5], can induce effective humoral immunity, but only weak cellular immunity, which is supposed to be beneficial for the prophylaxis and treatment of persistent HBV infection. Also, the current vaccination protocol recommends two to three doses to induce long-lasting immunity, and even after completion of the full HBV vaccine regimen, up to 10% of the population is unable generate a protective response to the virus [6]. The prevalence of S variant strains has increased in recent years [7]. Although currently used antiviral therapies, including treatment with pegylated interferon alpha 2a (PEG-IFN2α) or nucleos(t)ide analogues such as lamivudine [8], [9], significantly suppress HBV replication, they cannot completely eradicate the virus and can cause severe adverse reactions. Therefore, a therapeutic vaccine for chronic hepatitis B that enhances virus-specific immune responses and overcomes persistent HBV infection is urgently needed.

For the past 20 years, continuous efforts have focused on developing an effective therapeutic vaccine. These include the conventional prophylactic hepatitis B surface antigen (HBsAg)-based protein vaccines or a combination of vaccination with lamivudine antiviral treatment [10], [11]. Other strategies being explored include DNA vaccines designed to specifically stimulate HBV-specific T-cell responses [12], a multigene vaccine that contains five different plasmids encoding most HBV antigens and human IL-12 as a genetic adjuvant [13], and immunogenic complex (ICs) (HBsAg complexed with human anti-HBs)-based vaccines [14]. Some of these strategies have been demonstrated to reduce viremia and HBeAg/anti-HBe seroconversion and induce an HBV-specific T-cell response, but they could not achieve full control over HBV. Thus, further studies are still necessary.

Here, we explore other immune strategies to improve the HBV therapeutic immune response. Our preliminary data suggested that exposure of exogenous B- or T-cell epitopes to the virus-like particle (VLP) surface can enhance the immunogenicity of weak epitopes and can be built into a multivalent target antigen vaccine [15]. We constructed a protein vaccine HBSS1 containing S (1–223 aa) and PreS1 (21–47 aa), which can form stable, secreted VLPs with an equivalent molar ratio of PreS1 to S antigen [15]. PreS1 is a good vaccine candidate because the PreS1 region appears to play an important role in viral attachment to hepatocytes and in subsequent viral infectivity, and it is an efficient T- and B-cell immunogen [16], [17]. Moreover, previous studies have indicated that B-cell and T-cell epitopes are distributed throughout the preS1 region [18]. As a molecular carrier, the S protein has the unique property of self-assembling into 22-nm particles not only in mammalian cell lines but also in yeast [19]. We believe that the HBV surface (HBS) VLP can carry S1 epitopes and stimulate strong humoral and cellular immune responses, resulting in an effective HBV therapeutic vaccine.

The “prime–boost” regimen is a widely used vaccine strategy against many diseases, including AIDS, malaria, and cancer [20]–[22]. The vaccinia virus Tiantan strain is used as a vector because it is safe and can induce a strong immune response [23]–[26]. Previous studies have shown that HBV protein vaccines are not able to boost a functional antiviral cellular response [27], [28]; however, vaccines based on recombinant viruses have the ability to stimulate robust humoral and cellular immune responses [20]–[22]. In this study, we constructed a recombinant vaccinia (Tiantan strain) RVJSS1 that expresses the fusion protein SS1 and combined this with the HBSS1 protein vaccine to determine the optimal prime–boost regimen in C57BL/6 mice.

## Materials and Methods

### 1. Generation of vaccine candidates

The original vaccinia TianTan (TTV) and the dual-promoter insertion vector pJSA1175 were developed in our lab [25], [26]. The HBV(C genotype; subtype ayw), S (aa: 1–223), and PreS1 (aa: 21–47) fusion gene fragments, obtained from a Sal I -BamH I fragment of pVRC-HBSS1 [15] and treated with Klenow fragment, were cloned into the SmaI site of pJSA1175. The recombinant vaccinia virus RVJSS1 expressing the SS1 fusion protein was produced by transfection of pJSA1175-SS1 into chick embryo fibroblast (CEF) cells that were infected with TTV. The recombinant vaccinia virus RVJSS1 was then purified and propagated.

To confirm expression of the HBV antigens, CEF cells were infected with the recombinant vaccinia virus RVJSS1. The infected cells were maintained for 24 h at 37°C with 5% CO_2_ and were then fixed with 50% methanol. The HBV recombinant fusion proteins were detected using indirect immunofluorescence (IF) staining with rabbit antiserum against HBsAg. The level of the fusion proteins in supernatants and CEF cell lysates, harvested after 48 h, was determined by Western blot.

HBSS1 expressed in CHO cells containing S (1–223 aa) and PreS1 (21–47 aa) was constructed in our lab [27]. To determine whether the protein vaccine HBSS1 can form particles, we performed electronmicroscopy using negative staining as previously described [15]. The purity of HBSS1 was >95% and the antigen content was 100 µg/ml. The aluminium hydroxide [Al(OH)_3_] concentration used was 2 mg/ml.

### 2. Animals and immunizations

Female C57BL/6 mice 6–8 weeks of age (Animal Care Centre, Chinese Academy of Medical Science, Beijing) were randomly assigned into five groups (Table 1). Mice immunized with normal saline (NS) and the recombinant vaccinia virus RVJ were used as controls. The HBSS1 single dose (100 µl) was 1.25 µg, and the RVJ and RVJSS1 doses were 0.5×10^7^ pfu. Immunizations were given by intramuscular injection at the beginning of week 0 and at the end of week 3. All experiments were conducted in accordance with the Institutional Animal Care and Use Committee (IACUC) approved protocol.

**Table 1:** Total antibody positivity rate after single or double immunization.

Group	Vaccine or reagent		Anti-S		Anti-PreS1	
	Prime	Boost	Prime	Boost	Prime	Boost
1	NS	NS	0	0	0	0
2	RVJ	RVJ	0	0	0	0
3	RVJSS1	HBSS1	2/8	6/8	6/8	8/8
4	HBSS1	RVJSS1	5/8	7/8	8/8	8/8
5	HBSS1+Al(OH)3	RVJSS1	8/8	8/8	8/8	8/8

Note: Mice were randomly assigned to five groups (8/group). Except for the NS and RVJ mock groups, mice received different prime–boost vaccination regimens. Sera were collected 2 weeks after each immunization, and HBV specific antibody was detected by ELISA. Seroconversion is indicated in the table.

NS: Normal saline.

RVJ: recombinant vaccinia virus.

Serum samples were taken prior to priming and 2 weeks after each immunization for the study of PreS1- and S-specific antibody responses. The serum was isolated and stored at −70°C. Mice were sacrificed 2 weeks after the last immunization. The spleens of the mice were removed, and a single-cell suspension of splenocytes was prepared as previously described [15]. The final splenocyte preparations contained 2–5×10^7^ cells/ml in R10 medium (RPMI1640 medium with 10% FBS and 1% penicillin–streptomycin).

#### 2.1 ELISA

Mouse sera were tested for an HBV antigen-specific anti-S IgG antibody response using a commercially available enzyme-linked immunosorbent assay (ELISA) Kit (WanTai Co, Beijing)according to the manufacturer's instructions [15]. To detect the antigen-specific anti-PreS1 antibody, the microtiter plates coated with recombinant PreS1 protein were provided by the commercial diagnostic kit (Alpha, Shanghai, China). Briefly, 50 µl of serial dilutions (1∶10 to 1∶100,000) of mice sera were added to the wells and incubated for 1 h at 37°C. The plates were then washed and peroxidase-conjugated goat anti-mouse IgG (Sigma) was added and these plates were incubated for additional hour followed by a wash. A colour reaction was induced by adding 3,3,5,5-tetramethylbenzidine (TMB) peroxidase substrate solution and reaction was stopped by 2 M H_2_SO_4_. The absorbance at 450 nm was measured using an ELISA plate reader (Bio-Rad). The end titre was determined when the reading of the last serum dilution was two-fold greater than that in the negative control wells that contained mock mouse sera.

Determination of IgG subclasses was conducted as previously mentioned [28]. Serum samples from immunised mice were diluted at 1∶100, and the IgG subclass was determined using a murine antibody isotyping ELISA kit (Sigma). Biotinylated rat anti-mouse IgG1 (1∶1000) or biotinylated rat anti-mouse IgG2a and IgG2b (1∶2000) were used as controls.

#### 2.2 Synthesis and screening of HBV PreS1 and S peptides

According to the HBV PreS1 and S amino acid sequence(C genotype; subtype ayw) of HBSS1 vaccine, the HBV PreS1 relevant peptides (S1–5: DPAFRANTA; S1–6: RANTANPDW; S1–7: NPDWDFNPN; S1–8: NPNKDTWPD; S1–9: GFFPDHQLDPAFRANTANPDWDFNPNKDTWP) and the HBV S antigen relevant peptides (S1: VLQAGFFL; S2: IPQSLDSWWTSL; S3: FLGGTPVCL; S9: FILTRILTI) selected from amino acids 13–49 of S Ag, and S4 LLDYQGMLP, S5 GLSPTVWLS, S6 SILSPFIPLL, S7 VWLSVIWM, and S8 WGPSLYSIL selected from amino acids 97–215 of S Ag) were commercially synthesised using an Applied Biosystems 430A peptide synthesiser (Foster City, CA) and 9-fluorenylmethyl carbonate (Fmoc) chemistry. The peptides were purified using reverse-phase high-performance liquid chromatography (HPLC) to a purity of >95% and characterised by mass spectrometry. All peptides were dissolved in DMSO at 50 mg/ml and used at 5 µg/ml in experiments. An enzyme-linked immunospot assay (Elispot) was used to screen the optimal peptide for responses in C57BL/6 mice according to the manufacturer's instructions. Briefly, 96-well Multiscreen® Immobilon-P plates (Millipore) were coated with 5 µg/ml purified rat anti-mouse gamma interferon (IFN-γ) IgG1 (clone R4-6A2, BD Biosciences) in PBS, incubated at 4°C overnight and blocked for 2 h at 37°C. Individual peptides were added to the wells with 100 µl of freshly isolated splenocytes (5×10^5^ cells/well in R10 medium) in duplicate. The plates were incubated for 20–24 h at 37°C in 5% CO_2_. After washing, biotinylated detection antibody was added to wells, and plates were then incubated for 1 h at 37°C. After further washing, streptavidin–horseradish peroxidase was added for additional 1 h incubation at 37°C. After washing again, spots were revealed by adding AEC (3-amino-9-ethylcarbazole) substrate solution to yield a coloured spot after a 20–40-min incubation at RT in the dark. Finally, colour development was stopped by thoroughly rinsing with tap water. IFN-γ spot-forming cells (SFCs) were counted. The results are expressed as the number of SFCs per 10^6^ input cells. The number of peptide-specific IFN-γ-secreting T cells was calculated by subtracting the background (no-peptide) control value from the established SFC count.

#### 2. 3 ELISpot analysis of antigen-specific T cells

To evaluate the antigen-specific T cell responses induced by our prime–boost regimen, an IFN-γ ELISpot assay was performed. Freshly isolated mouse splenocytes were added at 5×10^5^/well with peptide pools. All the HBV S peptide pools and preS1 antigen relevant peptides synthesised were used; The HCV E1 peptide (SQLFTFSPRRYETI) was used as the nonrelevant negative control, PMA (50 ng/ml) and ionomycin (1 µg/ml) were added to the positive-control group, whereas the negative-control group received no added stimuli. All protocols were as described above.

#### 2.4 Intracellular cytokine staining (ICS) analysis

Approximately 0.5–2×10^6^ splenocytes from each immunization group were collected and cultured for 4 h at 37°C in 1640 supplemented with 10% FBS alone (unstimulated), or with 4 µg/ml HBV PreS1(S1–9) or S peptides pool (as above). All cultures contained Monensin (GolgiPlug; BD Pharmingen). After washing, cells were incubated for 30 min at 4°C with 25 µl of a 1/100 dilution of a PerCP -labelled Ab to mouse CD4 and FITC-labelled Ab to mouse CD8 (BD Pharmingen). The cells were washed again and permeabilised in 1× Cytofix/Cytoperm (BD Pharmingen) for 20 min at 4°C, washed three times with Perm/Wash (BD Pharmingen), and then stained with an APC-labeled anti-mouse IFN-γ or TNF-α mAb or PE-labeled anti-mouse interleukin-2 (IL-2) or interleukin-4 (IL-4) mAb (BD Pharmingen). Labelled cells were fixed in 1% formaldehyde-PBS. Samples were washed and analyzed by flow cytometer. Unstimulated T cells counts were used as background controls, and were subtracted when plotting data. Responses were considered positive when the percentage of total cytokine-producing cells was at least twice that of the background.

### 3 Statistical analysis

To compare the immune response between groups of experimental animals, statistical analyses were performed using ANOVA, an S-N-K test, and Pearson correlation analysis using SSPS 17.0. Results with a *p*-value <0.05 were considered statistically significant.

## Results

### 1 Construction, expression, and identification of recombinant vaccinia virus RVJSS1

**Figure 1:**
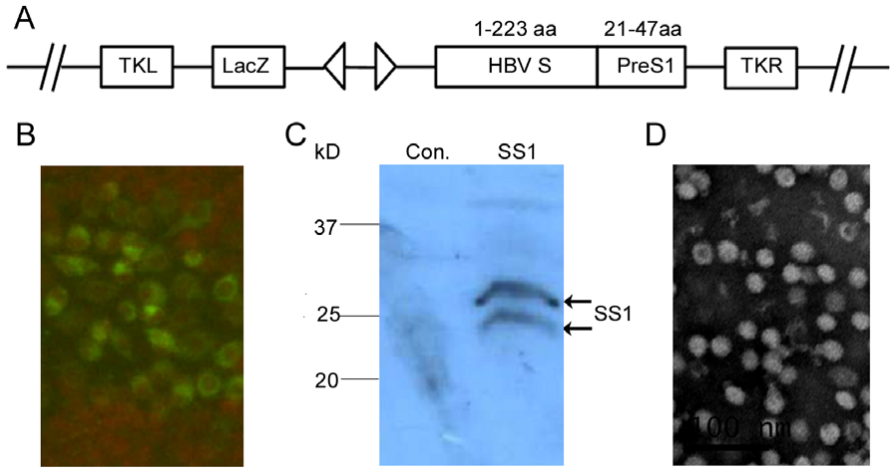
Characterization of recombinant vaccinia virus RVJSS1 and HBV particle-like subunit vaccine HBVSS1. (**A**) Schematic diagram of recombinant vaccinia virus RVJSS1, which contains two expression cassettes in a back-to-back orientation, flanked by vaccinia virus TK region sequences. Virus RVJSS1 was a Tiantan strain vaccinia virus with a *lacZ* gene led by a p11 promoter inserted into the TK region; the expression cassette on the right consists of the SS1 fusion protein led by the 7.5 K promoter. (**B**) Immunofluorescence assay to confirm SS1 fusion protein expression. CEF cells were infected by the purified RVJSS1 virus and fixed, permeabilised, stained with rabbit anti-PreS1 antibody and fluorescein isothiocyanate (FITC) conjugated to a secondary antibody, and then visualised using fluorescence microscopy. (**C**) Western blot analyses to detect expression of SS1 fusion proteins in CEFs infected with RVJSS1 using specific antibodies. The expression bands of the SS1 proteins are indicated by arrowheads. (**D**) Negative staining of purified HBVSS1 particles vaccine using electron microscopy.

The recombinant vaccinia virus RVJSS1 expressing HBV S and PreS1 fusion proteins was developed by homologous recombination in CEF cells with the shuttle plasmid pJSA1175-SS1 and the parent virus TTV. The resulting RVJSS1 was proved to be stable via low MOI(0.01 to 0.1) expansion in CEF cells and purified as previous described [25], [26]. Fig. 1A shows the structures of RVJSS1. Cells infected with RVJSS1 were analysed by IF assay and Western blot to confirm the effective expression of the two fusion antigens using S-specific antibodies for the HBV SS1 proteins (Fig. 1B, 1C). Electronmicroscopy also showed that HBVSS1(without alum salt) formed particles of diameter ∼22 nm (Fig. 1D).

### 2 Humoral immune responses of all prime–boost regimens

To optimise the prime–boost regimen, a series of prime–boost strategies was performed in C57BL/6 mice (Table 1). Serum samples were taken prior to priming and 2 weeks after each immunization. ELISA coating specific antigens (PreS1 and S) was used to determine whether seroconversion had occurred. No anti-S or anti-PreS1 antibody was observed in the NS and RVJ control groups; In the HBSS1+Al(OH)3+RVJSS1 group, the anti-S seroconversion rate was 100% (8/8) after prime immunization, which was higher than that of the RVJSS1+HBSS1 group (2/8) or the HBSS1+RVJSS1 group (5/8). For the anti-PreS1 antibody, the seroconversion rate for both the HBSS1+Al(OH)3 priming group and the HBSS1 priming group reached 100% (8/8), which was higher than that of the RVJSS1 priming group (6/8). After boost immunization, the anti-PreS1 antibody seroconversion rate of the RVJSS1+HBSS1 group also reached 100% (8/8), but the anti-S antibody seroconversion rates of the RVJSS1+HBSS1 and HBSS1+RVJSS1 groups were 6/8 and 7/8, which were lower than that of the HBSS1+Al(OH)3+RVJSS1 group (8/8). These results indicate that priming with HBSS1 or HBSS1+Al(OH)3 and boosting with RVJSS1 can accelerate seroconversion and increase the level of the antibody response.

**Figure 2:**
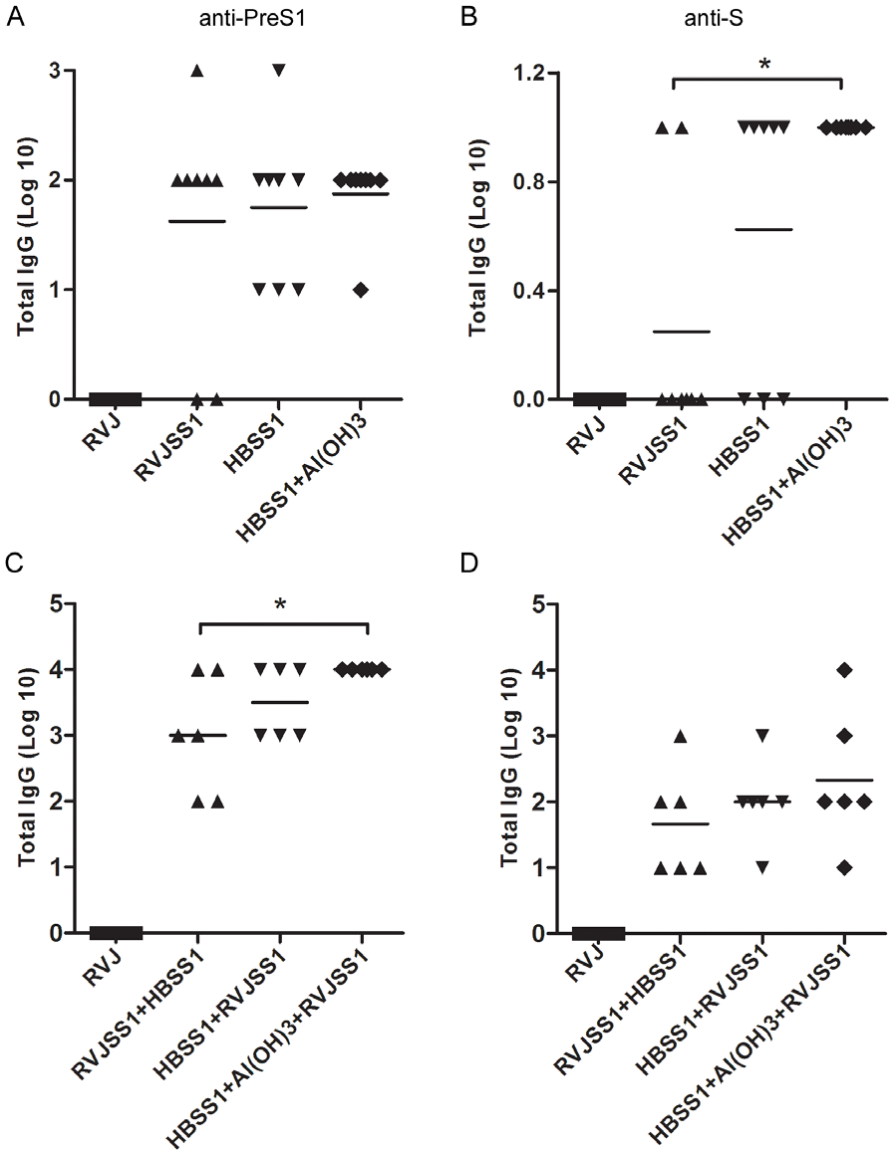
The anti-PreS1 and S antibody responses elicited by different regimens detected after first immunization (A–B) or second immunization(C–D). Each group received a different prime–boost vaccination, and antiserum was collected 2 weeks after the each immunization. Total HBV antigen-specific IgG titres were determined by ELISA. The symbols represent the titers of the sera from the individual mice. The horizontal lines represent the means (n = 6). The statistic significance of the results was analyzed and indicated as *p<0.05.

Specific antibody titres were determined by ELISA after each immunization. After priming immunization (Figure 2A and 2B), similar level of the anti-PreS1 antibody were elicited between RVJSS1 immunized group and HBSS1 immunized group. However,anti-S titres in group immunized with HBSS1+Al(OH)3 was significantly higher than RVJSS1immunized group. After the boosting immunization(Figure 2C and 2D), all immune groups revealed increased anti-PreS1 antibody and anti-S antibody titres than the priming immunization groups, and the average anti-S and anti-PreS1 antibody titres of the HBSS1+RVJSS1 and HBSS1+Al(OH)3+RVJSS1 groups were higher than those of the RVJSS1+HBSS1 group; Additionally the anti-PreS1 titer of HBSS1+Al(OH)3+RVJSS1 group were significantly higher than that of RVJSS1+HBSS1 group(*p*<0.05). Both anti-S and anti-PreS1 antibody titres of the HBSS1+Al(OH)3+RVJSS1 group were also slightly higher than those of the HBSS1+RVJSS1 group.

These data suggest that the HBVSS1 protein vaccine prime and recombinant vaccinia virus vector vaccine boost combination accelerated seroconversion and increased the level of the antibody response. Moreover, combination with aluminium adjuvant proved to be the best regimen, as it resulted in higher antibody levels and accelerated seroconversion.

### 3 IgG subtyping

**Figure 3:**
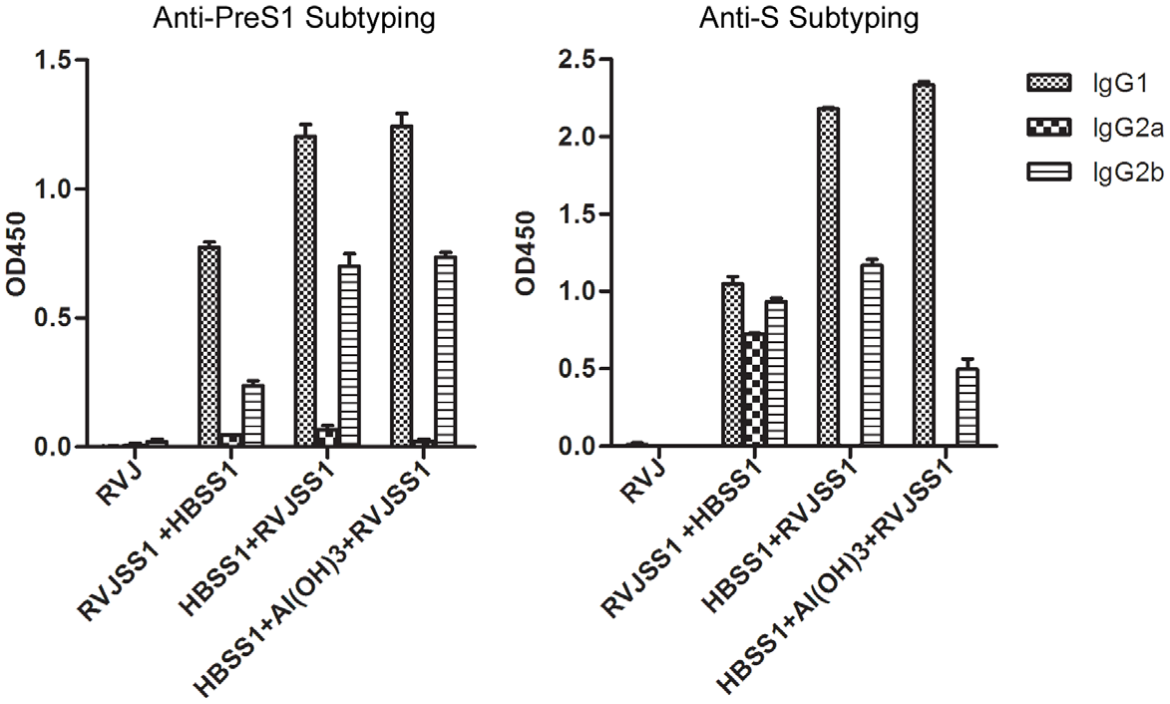
Subtype analysis of the HBV antigen-specific IgGs in sera of mice immunised with different vaccine combinations. Antigen specific IgG1 and IgG2a or IgG2b levels were determined using an IgG isotyping ELISA, as described in Materials and Methods. Sera were collected 2 weeks after the last immunization and diluted 1∶100. Bars indicate the average OD value at 450 nm (OD_450_) of each group.

The IgG subtype induced in each group was also evaluated by ELISA (Figure 3). Blood samples were collected 2 weeks after the last immunization and diluted 1∶100. The IgG subtypes of antibodies against PreS1 were primarily IgG2b and IgG1, whereas IgG2a levels were low in each group. There was a slightly greater amount of IgG1 compared with IgG2b antibodies against PreS1. Levels of both IgG2b and IgG1 antibody against PreS1 in the HBSS1+RVJSS1 and HBSS1+Al (OH)3+RVJSS1 groups were higher than that in the RVJSS1+HBSS1 group. The IgG subtypes of the antibodies against S in the RVJSS1+HBSS1 group were a mixture of IgG1, IgG2a, and IgG2b, whereas those in the HBSS1+RVJSS1 and HBSS1+Al (OH)3+RVJSS1 groups were mainly IgG2b and IgG1. The amount of IgG1 antibody against PreS1 and S in all groups was higher than that of IgG2b. In summary, all vaccine regimens induced a mixed Th1- and Th2-type immune response.

### 4 Identification of HBV PreS1 and S epitopes by IFN-γ ELISpot assay

**Figure 4:**
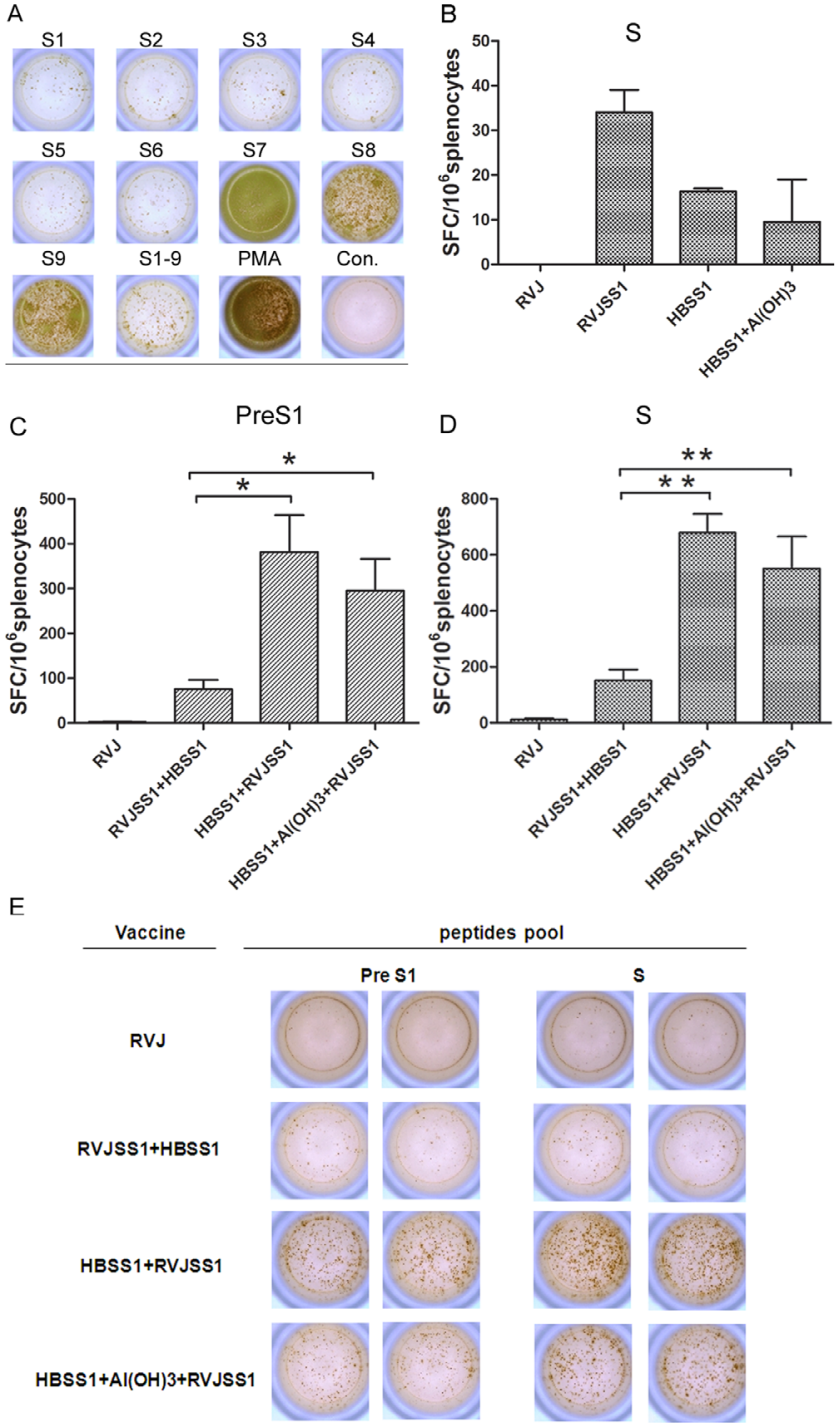
HBV S epitopes screening and ELISpot analysis of IFN-γ secretion in mouse splenocytes of each immunization group. (**A**) HBV S epitopes were screened by IFN-γ ELISpot analysis. Actual sample wells of HBS-specific ASC spots. (**B**–**D**) HBV peptide-specific ASC frequency in each group. Data represent the average of spot-forming cells (SFCs) per million splenocytes from six mice/group plus the standard error. **B**:Splenocytes were collected 10 days after the first immunization and the number of IFN-γ secreting cells generated in response to S peptides; **C–D**: Splenocytes were collected 2 weeks after the last immunization and the number of IFN-γ secreting cells generated in response to PreS1 and S peptide pool stimuli respectively. Statistical differences between groups were determined, and differences are shown as *p<0.05 and **p<0.01. (**E**) Sample wells with spots with mock or HBV peptide stimulation.

HBV PreS1 and S antigen-specific dominant epitopes for cellular-mediated immunity (CMI) in C57BL/6 mice were identified using IFN-γ ELISpot assays. Mouse spleen lymphocytes were stimulated with PreS1 or S peptide arrays as listed above. Mouse splenic lymphocytes stimulated by other peptide sections and non-stimulated mouse splenic lymphocytes were used to detect individual lymphocyte-colony IFN-γ secretion or its absence (Fig. 4A). The spots count from mouse splenic lymphocytes stimulated with PreS1 S1–9 peptides (30-mers, 12–42 aa of PreS1) and HBV S S7 (VWLSVIWM), S8 (WGPSLYSIL), and S9 (FILTRILTI) were significantly higher compared with lymphocytes stimulated with other peptide sections. These results indicate that we obtained three dominant HBV S antigen-specific epitopes in C57BL/C mice and PreS1 antigen-specific T-cell epitopes dispersed among the region around 21–42 aa.

### 5 Enhancement of the antigen-specific cellular immune response by the HBSS1 and HBSS1+Al(OH)_3_ prime and RVJSS1 boost regimen

To assess the cellular immune responses elicited by our prime–boost regimens, we next analysed the frequencies of IFN-γ-producing cells at the single-cell level by ELISpot assay. The peptide library used to stimulate the spleen cells was as described above. As shown in Fig. 4B, 10 days after prime immunization, the S-specific SFC readings of the RVJSS1 group were higher than that of the HBVSS1 and HBSS1+Al(OH)_3_ groups. Compared with the prime vaccination, the frequency of IFN-γ-producing cells in spleens from boost-immunised mice was dramatically increased. The number of IFN- γ secreting cells generated in response to PreS1 and S in the HBSS1+RVJSS1 and HBSS1+Al(OH)_3_+RVJSS1 groups was significantly greater than that in the RVJSS1+HBSS1 group (*P*<0.05) (Fig. 4C–D). Therefore, the protein-vaccine priming and vaccinia-virus-based vaccine boosting regimen significantly enhanced the antigen-specific cellular immune response.

**Figure 5:**
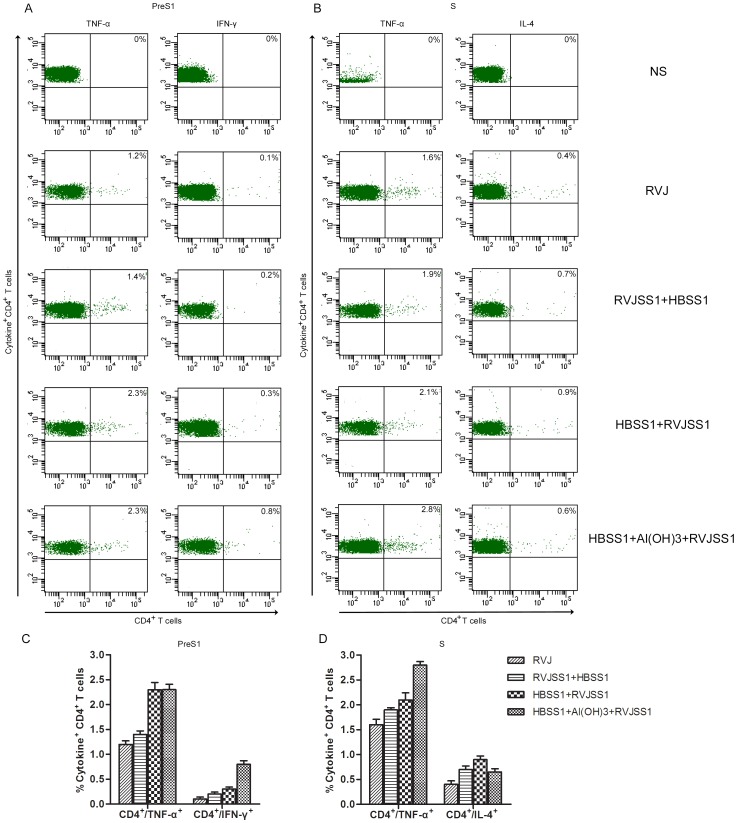
ICS analyse of HBV PreS1- or S-specific CD4+ cells producing interferon-γ (IFN-γ), tumour necrosis factor-α (TNF-α) and interleukin-4 (IL-4) induced by heterologous rTTV and recombinant protein prime/boost immunization. Splenocytes from four mice per group were isolated 14 days after last immunization. The splenocytes were exposed to HBV PreS1 peptide(S1–9) or S peptides pool and cytokine production was measured by monoclonal antibody staining and flow cytometric analysis. (a) Flow cytometer plot of results obtained from one representative individual mouse from each group. (b), Average (± SEM) of the percentage of IFN-γ-producing, TNF-α-producing and IL-4-producing CD4+ T cells obtained from four mice per group following stimulation with HBV PreS1 peptide (S1–9) or S peptides pool. Compared with RVJSS1 prime/HBSS1 boost, mice receiving HBSS1+Al(OH)3 prime/RVJSS1 boost generated markedly higher PreS1-specific CD4 T cell responses for two cytokines (IFN-γ and TNF-α, *P*<0.05) and S- specific CD4 T cell responses for TNF-α(*P*<0.05), mice receiving HBSS1 prime/RVJSS1 boost also generated markedly higher PreS1-specific CD4 T cell responses for TNF-α(*P*<0.05).

**Figure 6:**
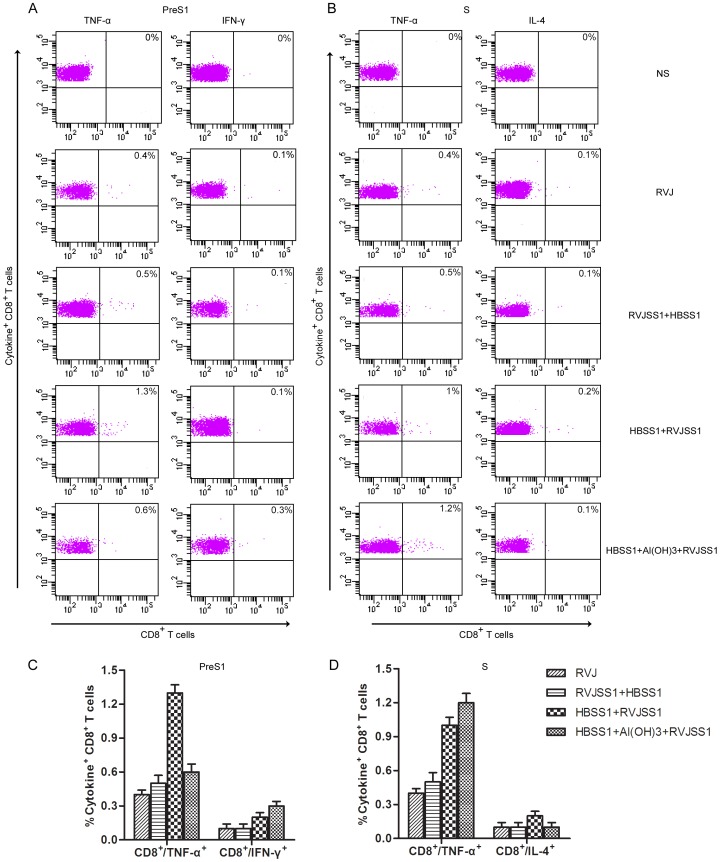
ICS analyse of HBV PreS1- or S-specific CD8+ cells producing interferon-γ (IFN-γ), tumour necrosis factor-α (TNF-α) and interleukin-4 (IL-4) induced by heterologous rTTV and recombinant protein prime/boost immunization. Splenocytes from four mice per group were isolated 14 days after last immunization. The splenocytes were exposed to HBV PreS1 peptide(S1–9) or S peptides pool and cytokine production was measured by monoclonal antibody staining and flow cytometric analysis. (a) Flow cytometer plot of results obtained from one representative individual mouse from each group. (b), Average (± SEM) of the percentage of IFN-γ-producing, TNF-α-producing and IL-4-producing CD8+ T cells obtained from four mice per group following stimulation with HBV PreS1 peptide (S1–9) or S peptides pool. Compared with RVJSS1 prime/HBSS1 boost, mice receiving HBSS1+Al(OH)3 prime/RVJSS1 boost generated markedly higher PreS1-specific CD8 T cell responses for IFN-γ(*P*<0.05) and S- specific CD8 T cell responses for TNF-α(*P*<0.05), mice receiving HBSS1 prime/RVJSS1 boost generated markedly higher PreS1- and S-specific CD8 T cell responses for TNF-α(*P*<0.05).

The Th1-cell and cytotoxic T lymphocytes (CTL) response to HBV and the associated secretion of antiviral cytokines (IFN-γ, TNF-α, and IL-2) may play a key role in virus clearance during HBV infection. ICS was therefore further used to assess HBV PreS1- or S-specific CD4+ and CD8+ T cells producing IFN-γ) TNF-α, IL-2, and IL-4 elicited by the protein prime/RVJ boost and vice versa. Subtracted with background controls, no HBV PreS1- or S-specific CD4+ and CD8+ T cells producing IL-2 were detected among immunized group (data no shown). The production of each of other three cytokines (IFN-γ, TNF-α, and IL-4) by either CD4+ or CD8+ T cells is shown in Figs. 5 and 6.

Compared with RVJ mock group, mice receiving RVJSS1 prime/HBSS1 boost generated only slightly higher HBV PreS1- or S-specific CD4+ T cell responses for TNF-α and S-specific CD4+ T cell responses for IL-4. However, compared with RVJSS1 prime/HBSS1 boost, mice receiving HBSS1+Al(OH)3 prime/RVJSS1 boost generated markedly higher PreS1-specific CD4 T cell responses for two cytokines (IFN-γ and TNF-α, P<0.05) and PreS1-specific CD8 T cell responses for IFN-γ(P<0.05), also S-specific CD4+ and CD8+ T cell responses for TNF-α (P<0.05). Mice receiving HBSS1 prime/RVJSS1 boost also generated markedly higher CD4+ and CD8+ T cell responses for TNF-α(P<0.05) than that receiving RVJSS1 prime/HBSS1 boost when in vitro stimulation with HBV PreS1(S1–9) or S peptides pool. However, PreS1-specific IL-4 and S-IFN-γ producing CD4+ and CD8+ T cells were undetectable in any group of this study (data no shown). Among above groups with heterologous prime/boost immunization, the strongest HBV S-specific CD4+ and CD8+ T cell responses for TNF-α was elicited in mice receiving HBSS1+Al(OH)_3_ prime/RVJSS1 boost. The strongest HBV PreS1-specific CD4+ and CD8+ T cell responses for TNF-α was elicited in mice receiving HBSS1 prime/RVJSS1 boost.

Taken together, these findings show that heterologous recombinant HBSS1 protein prime/RVJSS1 boost immunization are immunogenic and can generated CD4+ and CD8+ T cell responses for Th1 cytokines, particularly strong for TNF-α and IFN-γ.

## Discussion

HBV infection is a major public-health concern worldwide because of its association with chronic infection and progression to liver cirrhosis and hepatocellular carcinoma. Although strong antibody responses are important for clearance of and protection against HBV infection, the Th1-cell and CTL response to HBV and the associated antiviral cytokines (IFN-γ, TNF-a, and IL-2) may play a key role in virus resolution during natural infection [7], [29]–[30]. The ideal vaccine should be capable of eliciting strong humoral and cellular immune responses, especially Th1 and CTL responses. Therapeutic vaccination is a promising new strategy for controlling chronic infection. Some therapeutic vaccines such as the conventional prophylactic HBsAg based protein vaccines, lipopeptide-based vaccines, DNA vaccines, and others are under development, all of which have their own advantages and shortcomings as shown in the limited trials in human, chimps or other animal models [2], [7]. Thus an optimised immunization regimen is a promising strategy for controlling HBV infection.

The presence of both humoral and cellular mediated immunity is important for protection and clearance of HBV infection. In this study, we used a well-described subunit protein (HBSS1) and recombinant vaccinia virus (rTTV) based-vaccine (RVJSS1) candidate, which is acceptable for clinical use. We found that an HBSS1 priming and RVJSS1 boosting strategy induced humoral and cellular immune responses to HBV in C57BL/6 mice. We chose a S-PreS1 fusion as the target antigen for the novel HBV vaccine candidate, because the HBsAg PreS1 region correlates with the assembly and infectivity of HBV, possesses a site for binding to hepatocyte membranes, and has abundant T- and B-cell epitopes. This may therefore overcome the lack of response to common vaccines containing only the S region [15]–[17]. A new study found that the neutralizing epitopes in the preS1 attachment site of hepatitis B virus lie in the N terminal (2–48), and addition of the preS1 (2–48) peptide in a highly immunogenic form to the current hepatitis B vaccine may improve protective immunity and reduce selection of escape mutations [31]. Moreover, incorporation of the preS1 region into epitope-based vaccines is widely accepted. We previously constructed a particle-like structure expressing plasmid pVRC-HBSS1, in which the PreS1 (21–47 aa) gene was fused to the C-terminal of the S (1–223 aa) gene of HBV, resulting in generation of strong cellular and specific S1 and S antibody responses. Furthermore the PreS1 antibody was indeed produced earlier than the S antibody and favoured a Th1-type response [15]. In this study, both the protein-particle vaccine HBSS1 that derived from CHO system and the recombinant vaccinia virus (Tiantan)-based vaccine RVJSS1 consisted of a S and a PreS1 fusion antigen, and higher S or PreS1-specific antibody levels as well as specific cellular immune responses (IFN-γ ELISpot analysis) detected in all vaccination groups. The immune responses in groups primed with HBSS1 or HBSS1+Al(OH)_3_ and boosted with RVJSS1 were significantly higher than those of the RVJSS1 prime and HBVSS1 boost groups. All immune responses were a mixed Th1 and Th2 type.

Recombinant protein vaccines induce mainly antibody responses but are not favorable to boost a functional antiviral T-cell response [2]. However, an HBV therapeutic vaccine should include activation of CD4+ T cells with a Th1 bias to generate anti-viral cytokines and promote CD8+ T-cell-mediated clearance of virus through cytolytic and non-cytolytic (anti-viral cytokines) activities [7]. Thus, therapeutic immunization using recombinant viral vectors and a prime–boost strategy may be a good choice. Viral vectors offer a series of advantages over traditional vaccines; in addition to inducing outstanding antibody responses, they also elicit CTLs that are crucial for control of intracellular pathogens and cancer and that are not induced by protein-based vaccines [32]. Recently, protein vaccine and viral vector prime–boost regimens have been used in vaccines against various human pathogens [20], [22], [32], [ and 33]]. The Thai efficacy trial of a prime–boost regimen comprising a canarypox vector (ALVAC-HIV, Sanofi Pasteur) followed by a gp120 subunit in Alum (AIDS VAXB/E, Global Solutions for Infectious Diseases) showed a statistically significant trend towards preventing HIV infection in an at-risk population [33]. Also, a malaria pre-clinical study found that the combination of protein in adjuvant with viral vectors in both priming and boosting vaccinations could induce extremely high antibody titres and CD4^+^ T-cell and CD8+ T-cell responses [34]. In this study, we tested three protein prime–boost regimens and found that the specific cellular immune responses (IFN-γ ELISpot analysis) in the RVJSS1-primed group were slightly higher than those in the HBSS1- and HBSS1+Al(OH)_3_-primed groups. However, after boost vaccination, the cellular immune responses of the groups boosted with RVJSS1 were significantly higher than that of those boosted with HBSS1. Thus, the recombinant vaccinia virus was critical for generation of cellular immune responses. Compared with our previous study, the protein-vaccine prime and recombinant vaccinia-virus-boost strategy induced both higher S and S1 antigen-specific SFC readings than did DNA priming and protein boosting (pVRC-HBSS1+HBSS1+Al(OH)_3_) [35] or homologous immunization (HBSS1+Al(OH)_3_+HBSS1+Al(OH)_3_) [28]. This was particularly the case for the S1 antigen-specific SFC readings of 300–400 SFC/10^6^ spleen cells compared with fewer than 50 SFC/10^6^ spleen cells for the other two strategies. Additionally, with the exception of the pVRC-HBSS1+HBSS1+Al(OH)_3_ groups (IgG1/IgG2 <1), all groups exhibited IgG1/IgG2 values >1 [28], [35]. Our data is also consistent with previous report indicated that both enhanced Ag-specific T cells immunity and Abs could be induced in BALB/c mice by combination vaccine of HBV subunit protein (comprising S and PreS2-Ags) and recombinant poxviruses (MVA) [36]. Therefore, an HBSS1 protein prime and RVJSS1 virus-boost strategy would be the most promising regimen for the future HBV therapeutic vaccination.However, the potential impact of pre-existing immunity should be further explored if this regimen applying for human.

In our prime–boost regimen, we choose Al(OH)_3_ as an adjuvant; this is also used in traditional HBV subunit vaccines. Although Alum alone is well known to activate Th2 responses, it might be enable to activation of CD4 T cells with a Th1 bias when combination with other adjuvant (CpG) or vaccine (viral vector,DNA) in prime boost vaccination as reported by several study. And the role of strong Ab response in the HBV therapeutic vaccine might not be excluded currently. We discovered that the anti-S antibody positivity rate of the HBSS1+Al(OH)_3_ group was higher than those of the RVJSS1 and HBSS1 prime groups; these results are similar to those of other studies that reported induction of Th2-type immune responses and increased production of protective anti- HBs antibodies [2]. Both HBSS1 subunit priming groups exhibited higher anti-S1 antibody positivity rates than did the RVJSS1 primed group. When specific IgG isotype were assayed among the prime-boost group, mice primed with RVJSS1 produced substantially higher IgG2a except IgG1 and IgG2b isotypes, while mice primed with subunit vaccine (HBSS1) produced significant higher titer of IgG1 and comparable IgG2b but no IgG2a. Our previous data indicated that subunit vaccine (HBSS1) apply alone produced dominantly IgG1 isotype while RVJSS1 alone produced dominantly IgG2a and IgG2b isotypes (no shown). We might conclude that IgG subclass or Th biased immune response elicited by prime-boost immunization might be dominantly modulated by Ag priming.

T cell epitope mapping was also tested by ELIspot in C57 BL/6 mice (H-2^b^) after heterologous prime-boost immunization (Figure 4A). It is somewhat surprising that C57BL/6 mice (H-2^b^) not only displayed a response to the well-described Kb-restricted S7 peptide but also to a D^d^-restricted peptide (S8) and to an HLA-A2-restricted epitope (S9). This cross-presentation or cross-priming phenomenon may be induced by the combination heterologous vaccines and prime-boost approach, which might enhance the magnitude and breath of MHC class I Ag processing [37], [38], but need to be further demonstrated.

A series of studies in HBV transgenic mice and HBV-infected chimpanzees revealed that HBV specific CD8+ T cells activated by immunization or infection were shown to produce cytokines, such as IFN-γ and TNF-α, which suppressed HBV gene expression and replication without destroying the infected hepatocytes [39], [40]. ELISpot assay data in this study showed that the protein-vaccine priming and vaccinia-virus-based vaccine boosting regimen significantly enhanced the antigen-specific cellular immune response compared to the vice versa when analysed the frequencies of IFN-γ-producing cells. Furthermore, ICS for Ag-specific CD4+ and CD8+ T cells producing cytokines confirmed that HBSS1 protein prime/RVJSS1 boost immunization are highly immunogenic and can generated more significant level of both CD4+ and CD8+ T cell responses for Th1 cytokines(TNF-α and IFN-γ). No significant liver pathogenic change after immunization was observed in our mice model (data no shown).We might conclude that the HBSS1 protein-vaccine prime and RVJSS1 boost immunization is one of the most promising approaches of therapeutic vaccination against HBV infection.

In conclusion, we found that the HBV protein-vaccine prime and recombinant vaccinia (Tiantan)-based vaccine boost strategy induced the highest humoral and cellular immune responses and generated significant increase of both CD4+ and CD8+ T cell responses for Th1 cytokines, particularly TNF-α and IFN-γ. Although C57BL/6 mice cannot be used to assess the HBV load attenuation as HBV transgenic mice model, they are still useful in the test of therapeutic immunity since reactivation of HBV multi-antigen specific immune cells and induction of polyfuntional T –cell responses might be the ultimate goals of HBV immune therapy. However, further studies aimed at developing a good small-animal model to evaluate vaccine efficacy and/or testing the vaccine in a large-animal model are needed. Since an HBSS1 priming and RVJSS1 boosting strategy induced strong HBV Ag-specific humoral and cellular(cytolytic CD8 and perhaps CD4 T cell) immune responses in mice, which is an attractive option in the design of therapeutic HBV vaccines [2], [7], [29]. In addition, significant IFN-γ and TNF-α induction in this regimen were the two cytokines that were found to mediate CD8+ T cell antiviral activity in the HBV-transgenic mouse model and in experimental HBV infection of chimpanzees [39], [40], our results may pave a way for the rational design and application of therapeutic vaccination against HBV infection.
